# High-speed data transmission over a microresonator frequency comb with dispersion compensation for augmented data rates and reach

**DOI:** 10.1515/nanoph-2023-0940

**Published:** 2024-03-22

**Authors:** Kenny Y. K. Ong, Aadhi Abdul Rahim, Xavier X. Chia, George. F. R. Chen, Peng Xing, Dawn T. H. Tan

**Affiliations:** 233793 Singapore University of Technology and Design, Singapore, Singapore; A*STAR Institute of Microelectronics, Singapore, Singapore

**Keywords:** dispersion compensation, high-speed data, microresonator frequency comb, silicon photonics

## Abstract

Microresonator frequency comb-based high-speed data transmission provides a pathway towards augmented data capacity without increasing the number of laser sources. Their use with intensity-modulated direct detection modulation (IMDD) formats is especially pertinent in data center communications where minimizing cost, latency and complexity is paramount. This however implies that the same extent of digital signal processing techniques commonly used in coherent detection for the management of fiber impairments including chromatic dispersion are not available. With the proliferation of silicon photonics technologies in data center transceivers integrated dispersion compensation which can overcome fiber impairments would be of great merit. We demonstrate low power generation of the primary comb state in a silicon nitride microresonator and transmission of 25 Gb/s NRZ and 50 Gb/s PAM4 data over 20 km of single mode fiber. This represents the longest fiber reach demonstrated to date for the transmission of IMDD data using an integrated, microresonator frequency comb. An integrated, tunable grating device for dispersion compensation that reduces dispersion impairments after several fiber lengths generates significant improvements in the eye diagram, six orders of magnitude improvement in the bit-error rate and 14 dB improvement in the transmitter and dispersion eye closure quaternary values. Concurrently, doubling data transmission is demonstrated via polarization multiplexing a comb line and successful dispersion compensation of up to 20 km.

## 1 Introduction

The rapid development in data transmission links using advanced modulation formats and multiplexing techniques have expanded channel capacities beyond 150 Tb/s [1], [2], [3]. Additionally, the compact on-chip WDM (wavelength division multiplexing) channels from integrated microresonator frequency combs are experiencing accelerated growth in high-speed data communications [4], [5], [6]. In particular, frequency comb-based high-speed data provides a low-cost, highly coherent multi-wavelength source, which has received significant attention in recent days. The notable recent developments in this domain include 55 Tb/s aggregate data transmitted over 75 km using QPSK and QAM modulated signals [7], and 44.2 Tb/s transmission using a soliton crystal [8]. These coherent modulation formats are typically used for long-haul high speed data transmission, where the availability of digital signal processing (DSP) helps to ameliorate impairments from the optical fiber [9], [10]. Even though coherent modulation techniques are efficient for higher data rates, the system’s complexity, cost, and high latency make it less suited for data center communications and data-intensive applications [11], [12].

On the other hand, due to its simplicity, low cost, and low power consumption, intensity modulated direct detection (IMDD) modulation formats largely dominate in optical communication systems for data transmission. To augment data rates, higher order IMDD modulation such as PAM4 or PAM8 is used to double or quadruple the symbol rate compared to the conventional non-return-to-zero (NRZ) data signal. Similarly, the compact on-chip IMDD data over multiple frequency comb lines are suitable for higher data transmission rates with significant increase in data capacity over the optical link. Despite all these improvements, the optical fiber links for long-distance communications and passive optical networks (PONs) still encounter significant challenges due to distortions in the data signal caused by group-velocity dispersion (GVD) and nonlinearity. Therefore, the high-speed data transmission through the single mode optical fiber link limits the reach and rate of the transmitted data. The limitation in the transmission of high-speed IMDD data over frequency comb lines is also subject to fiber dispersion which results in a reduction in the bit-error rate. This is particularly noticeable when adopting advanced modulation formats which have a lower signal to noise ratio, especially at higher data rates and longer fiber reaches. Typically, the signal degradation due to GVD is managed through two approaches, either by employing a dispersion compensating fiber (DCF) having an opposite GVD profile or using DSP algorithms. The DSP-based GVD compensation is computationally expensive, leading to a bulky setup with high power consumption. On the other hand, DCFs typically require long fiber lengths on the order of tens of kilometers with fixed dispersion, leading to latency and undesired nonlinear effects. Therefore, the implementation of tunable dispersive lines in a compact on-chip integrated platform [13], [14], [15] suitable for multichannel frequency comb-based transmission link would be of great merit.

In this paper, we present a fully integrated SiN platform design, incorporating a micro-ring resonator-based multi-wavelength light source and ultralow-loss grating-based on-chip dispersion compensation element for high-speed data transmission over fiber. We demonstrate the successful transmission of 25 Gb/s NRZ and 50 Gb/s PAM4 data over 20 km of optical fiber, utilizing modulation on the integrated microresonator frequency comb in conjunction with a 4.2 mm long on-chip, integrated dispersion compensation device. The comb lines are demultiplexed and modulated with NRZ and PAM4 data before traversing through fiber lengths of up to 20 km. We achieve error-free transmission of 50 Gb/s PAM4 data modulated over the microresonator frequency comb. Significant improvement in the eye diagram and up to six orders of magnitude reduction in bit-error rates (BER) are achieved using an integrated dispersion compensation device, greatly facilitating the transmission of high-speed data modulated on Kerr frequency combs over long fiber reaches. Lastly, we show that the TE and TM mode of the grating device can be utilized to compensate for dispersion from two orthogonal polarization axis from three different resonances accessed from the micro-ring resonator.

## 2 Device characterization of the integrated micro-ring resonator and grating devices

**Figure 1:**
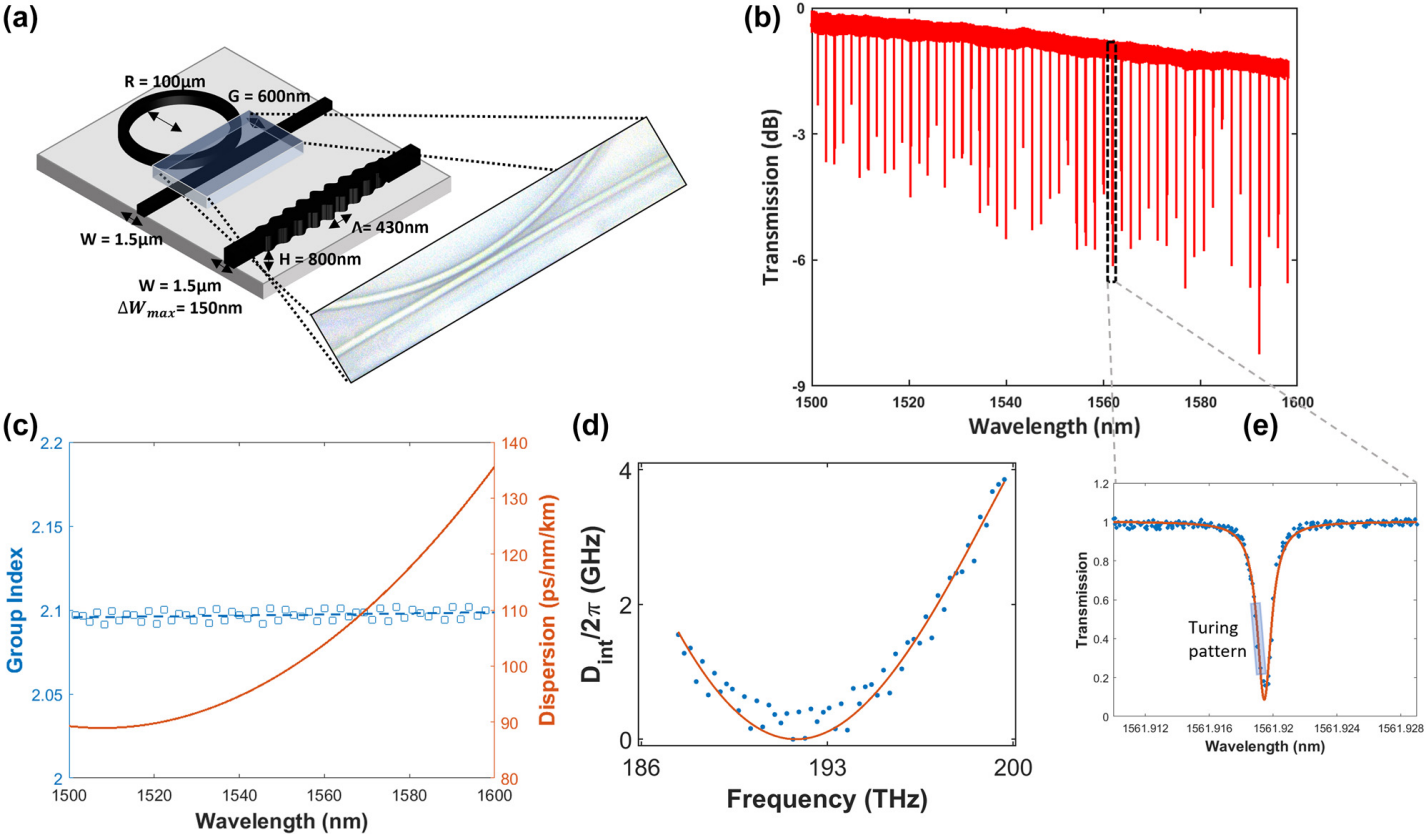
Characterization of the integrated micro-ring resonator for frequency comb generation. (a) Schematic of the integrated micro-ring resonator and grating device for dispersion compensation. (b) Transmission spectrum of the resonator with (e) the region where Turing patterns are generated in a normalized transmission spectrum with a Lorentzian fit. (c) Measured group index (left) and dispersion (right) of the micro-ring resonator as a function of wavelength. (d) The intrinsic dispersion of the micro-ring resonator as a function of frequency (THz).

A silicon nitride (SiN) micro-ring resonator is used to generate the frequency combs. The resonator has a radius of 100 μm, a width of 1.5 μm and a gap of 600 nm between the ring resonator and bus waveguide. The SiN core is fabricated with a height of 800 nm and surrounded by SiO_2_ cladding. The dimensions of the two devices on a single silicon nitride chip are shown in Figure 1a and the inset shows an optical micrograph of the waveguide-ring region showing the 600 nm gap. Figure 1b shows the transmission spectrum of the resonator with an FSR of ∼2 nm. We generate the Turing patterns (primary comb state) by pumping resonances located in the C and L band (1525 to 1600 nm).

Figure 1e shows the region with a pump resonance of 1560 nm to generate a primary comb state by performing a wavelength scan from the blue side of the resonance and stopped before secondary side combs appear, near the trough of the resonance, where the individual frequency comb lines have maximum peak power and the sidemode-to-pump ratio is lowest [16]. The advantage of using the primary comb state is that it does not require feedback and active stabilization, displaying stronger phase-locking characteristics as compared to solitons [17], [18], which would result in lower BERs after modulation of the frequency comb line.

Figure 1c shows the group index that was used to calculate the dispersion (ps/nm/km) from the micro-ring resonator as a function of wavelength. It is observed that the micro-ring resonator exhibits anomalous dispersion across the C and L band, important for phase matching involved in the generation of the primary comb state. Lastly, from Figure 1d, it shows the intrinsic dispersion centered at 1560 nm, that was calculated from the following: *ω*_*μ*_ − *ω*_0_ = *D*_1_ *μ* + *D*_2_ *μ* ^2^/2 + *D*_3_ *μ* ^3^/2 = *D*_1_ *μ* + *D*_int_(*μ*) where *ω*_*μ*_ and *ω*_0_ are the resonance location and center resonance location, respectively [19]. With *D*_1_, *D*_2_ and *D*_3_ representing the free spectral range (FSR), group velocity dispersion (GVD) and higher order dispersion terms, Figure 1d shows that the magnitude of intrinsic dispersion ( $\left.D_{\text{int}}\right)$ , which is the deviation of a resonance from an equidistant frequency grid, is low in the C and L bands (0 to 4 GHz).

After modulation of a single frequency comb line with NRZ/PAM4 data, the optical field propagates through a standard single mode fiber (SMF), incurring a characteristic fiber dispersion of 16 ps/nm/km at 1550 nm and dispersion provided by the grating device compensates for these dispersion impairments. Here, we used a separate on-chip SiN grating device for dispersion compensation. Note that this configuration is equivalent to dispersion compensation at the receiver end of a transceiver link.

**Figure 2:**
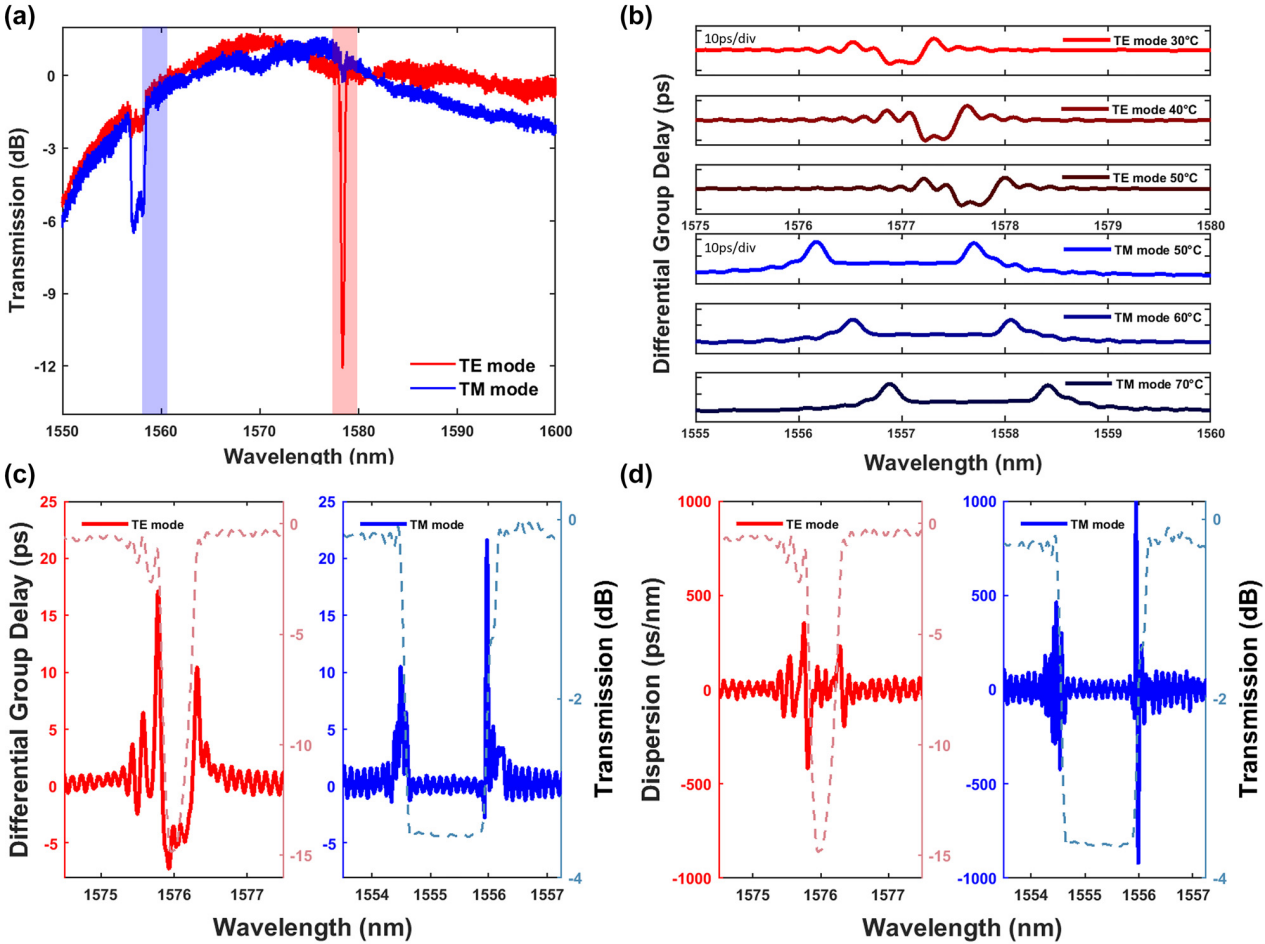
Characterization of the integrated grating device for dispersion compensation. (a) Transmission spectrum of a single grating device where the TE mode (red) and TM mode (blue) can be used for dispersion compensation. The highlighted region shows the operating wavelength range used for dispersion compensation in this work. (b) The measured group delay spectrum for the TE (red) and TM (blue) mode measured as a function of temperature. (c) The differential group delay (ps) and (d) dispersion (ps/nm) spectrum for the TE and TM modes. Dashed lines indicate the transmission spectrum of the TE and TM modes.

A grating on the same silicon nitride chip was used for dispersion compensation with a grating pitch (Λ) of 430 nm, length (*L*), width (*W*), and height (*H*) of the gratings are 4.2 mm, 1.5 μm, and 800 nm, respectively. A raised cosine apodization was used to reduce the Fabry–Perot oscillations at the edge of the bandgap by gradually increasing the sinusoidal sidewall modulation amplitude from the ends of the grating to a maximum of Δ*W* = 150 nm at the center of the grating. Figure 2a shows the transmission spectrum of the dispersion compensation device tuned to the TE (red) and TM modes (blue), by using a polarization controller before transmission through the dispersion compensation device. The highlighted regions indicate the regions where the device was thermally tuned to, to access the required normal dispersion magnitude for dispersion compensation of a comb line source.

As the frequency comb lines are fixed, and because different fiber lengths require different magnitudes of normal dispersion, the dispersion compensation device is thermally tuned to access the dispersion magnitudes needed for each fiber length. Silicon nitride has a thermo-optic coefficient of $\left.2.45\pm 0.09\right)\times 10^{-5}\left(\frac{\text{RIU}}{{}^{\circ}\text{C}}\right)$ [20]. With the thermo-optic effect, we used a Peltier module with a temperature controller to thermally tune the grating device with a temperature resolution of 0.001 °C. Thermally tuning the grating device creates a wavelength shift and shifts the group delay spectrum as shown in Figure 2b, where there is a red shift in the group delay spectrum for both modes as temperature increases with a wavelength shift of 34 pm/°C. The Bragg wavelength measured at 20 °C for one of the grating devices for the TE and TM modes were near 1577 nm and 1556.3 nm, respectively.

To eliminate dispersion impairments, the accumulated dispersion after transmission through the SMF must ideally be met with an equal magnitude of normal dispersion that is proportional to the length of the SMF. As shown in Figure 2c, the group delay $\left(\frac{\partial \varphi }{\partial \omega }\right)$ increases near the band edge and as dispersion is the derivative of the differential group delay $\left(\frac{\partial^{2}\varphi }{\partial^{2}\omega }\right)$ , the magnitude of dispersion subsequently increases. Near the maximum group delay, the magnitude of dispersion also reaches a maximum. Thermo-optic tuning to access the required dispersion magnitude near the maximum group delay from both TE and TM response of the device still allows a low latency with less than 23 ps in group delay to be achieved. On the red-side of the bandgap in the TE mode, as shown in Figure 2d, a sufficient magnitude of normal dispersion can compensate for 4 km (−64 ps/nm), 6 km (−96 ps/nm) and 10 km (−160 ps/nm) of SMF. However, a larger magnitude of normal dispersion can be accessed on the blue-side of the bandgap that is sufficient to compensate for dispersion from 20 km SMF (−320 ps/nm), albeit requiring a slightly higher temperature for a further redshift. For the TM mode, the blue side of the bandgap has a sufficient magnitude of normal dispersion to compensate for dispersion from a 20 km SMF. To compensate for the frequency comb line at 1577.12 nm for a pump resonance at 1560 nm, the TE mode of the dispersion compensation device was utilized such that a temperature range of 36–40 °C was needed to compensate for fiber lengths of 4 km, 6 km, 10 km, and 20 km. As for the TM mode, the temperature was about 75 °C to compensate for 20 km of optical fiber.

In this work, we show dispersion compensation demultiplexing (1) with polarization demultiplexing of a comb line source using the TE and TM modes of the dispersion compensation device to compensate for 20 km of SMF and (2) of augmented fiber lengths (6 km, 10 km, and 20 km) with PAM4 data signals and (3) across comb lines.

## 3 Methods

**Figure 3:**
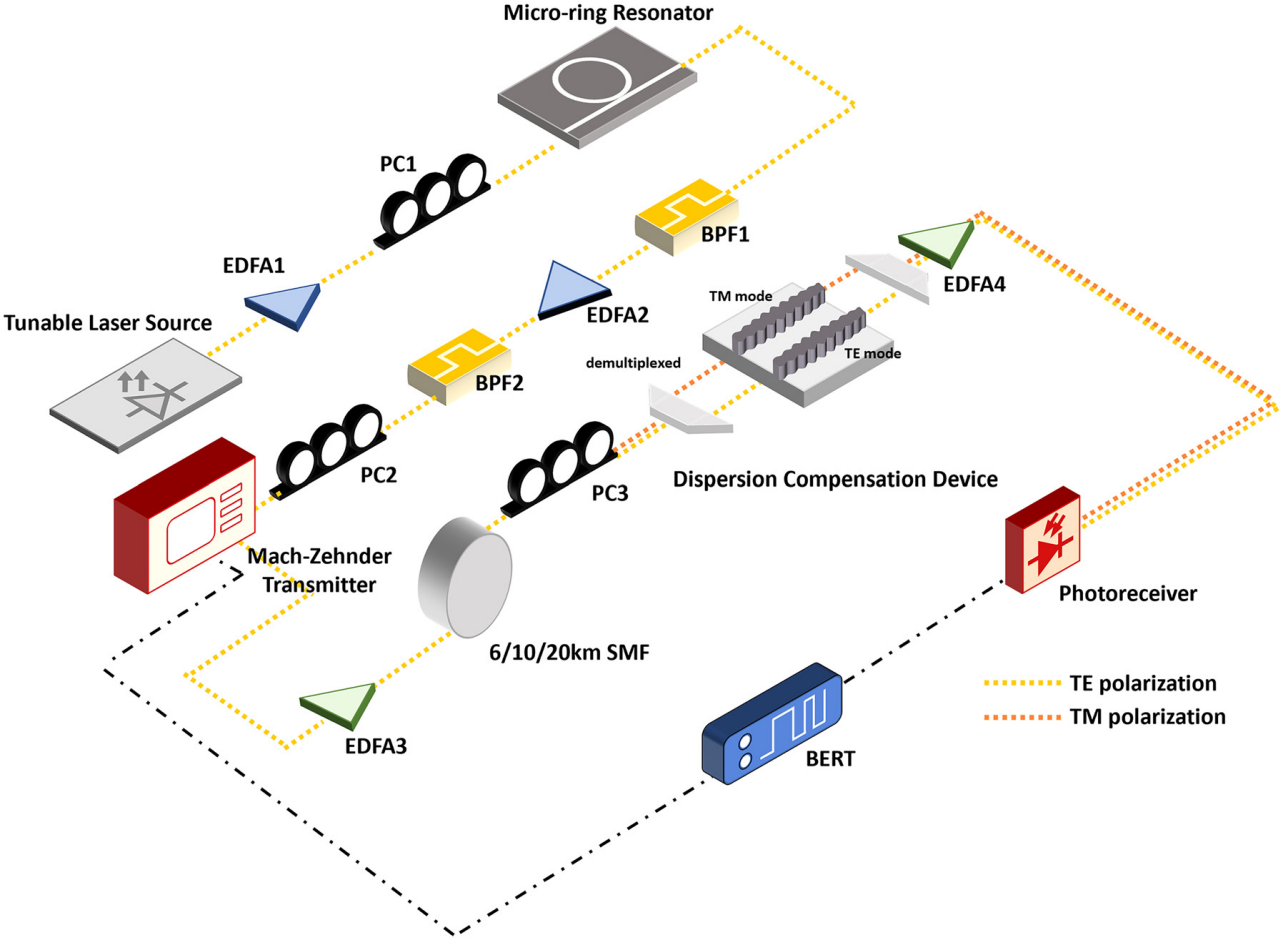
Schematic of the generation of the frequency combs and high-speed measurements of the modulated comb line with dispersion compensation. EDFA – erbium doped fiber amplifier, PC – polarization controller, BPF – bandpass filter, SMF – single mode fiber, BERT – bit error rate tester.

Figure 3 shows the schematic of the setup used for the high-speed measurements. A tunable continuous-wave laser source is used to access the targeted resonance from the micro-ring resonator. A pump power of 150 mW was used to generate the frequency comb lines in the primary comb state. A polarization controller is used to optimize the polarization that is coupled into the micro-ring resonator device. The generated frequency comb is transmitted through a bandpass filter (BPF), and then amplified by an erbium-doped fiber amplifier (EDFA) before being transmitted through another BPF to isolate a single frequency comb line. This configuration is optimized to imitate closely a reference laser source, where the amplification noise from the EDFA is removed, resulting in a lower SNR while maintaining a high power. A polarization controller is also used before the transmitter to ensure optimum polarization control. The filtered single frequency comb line is modulated with a Mach–Zehnder interferometer and a fixed RF gain for each individual comb line was used in the experiments. A bit error rate tester (BERT) generates 25 Gb/s NRZ and 50 Gb/s PAM4 data signals with a pseudo-random binary sequence (PRBS) with 31 patterns (2^31^-1 bits) and 13 patterns (2^13^-1 bits) respectively. The modulated signal is further amplified with an EDFA and then transmitted through either 4 km, 6 km, 10 km, or 20 km of single mode fiber (SMF). A polarization controller is used before propagation through the dispersion compensation device, to tune and maximize to the TE or TM mode of the grating device (color referenced as shown in Figure 3). A feed-forward equalization (FFE) was performed for the back-to-back configuration to resolve electrical impairments from the E-O-E conversion and an all-optical process was used in the experiments without other equalization processes or forward error correction (FEC). After propagating through the dispersion compensation device, the data signal is received by a PIN-TIA photoreceiver. Dispersion compensation from the grating device reduces these dispersion impairments.

## 4 Frequency comb generation and high-speed measurement

### 4.1 Dispersion compensation of polarization demultiplexed data signal

**Figure 4:**
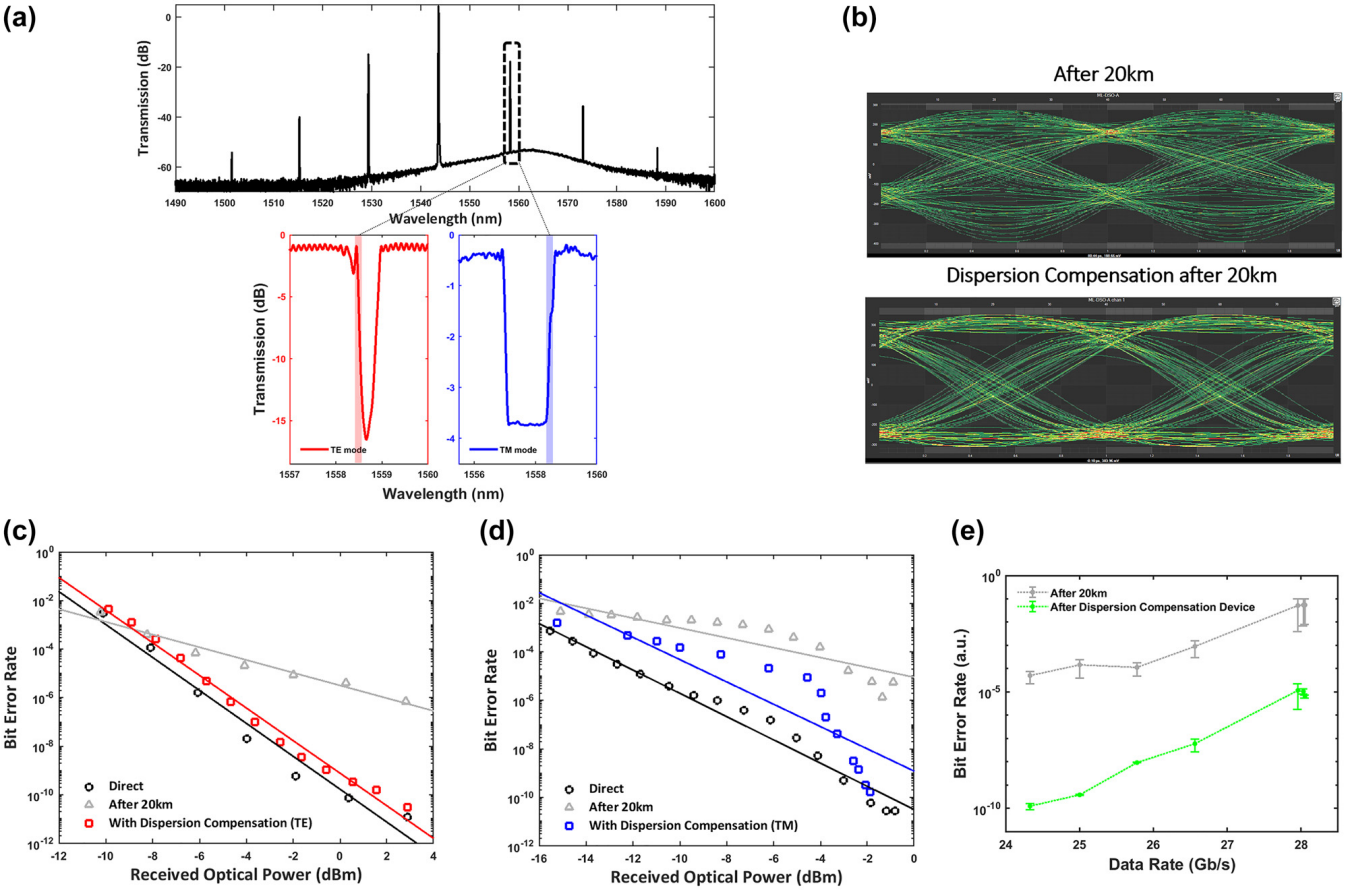
High-speed measurements and dispersion compensation of a polarization demultiplexed data signal. (a) Frequency comb spectrum (above) generated by pumping at the microresonator resonance of 1545 nm. The transmission spectrum of the TE and TM modes (below) are thermally tuned such that the highlighted region of the operating wavelength is aligned to the frequency comb line source to access the magnitude of dispersion required for compensation. (b) Eye diagram comparison with 25 Gb/s NRZ data signals transmitted after 20 km and after the dispersion compensation device. Bit-error rate as a function of received optical power showing dispersion impairments after 20 km and after dispersion compensation in the (c) TE mode and (d) TM mode. (e) Bit-error rate as a function of data rates (Gb/s) after 20 km and after the dispersion compensation device using the TE and TM modes.

Firstly, to show dispersion compensation using two modes of the grating device, the CW laser was tuned into the resonance of 1545 nm to generate the frequency combs. Utilizing another orthogonal polarization axis, as is often used in polarization division multiplexing, also increases the amount of data that can be transmitted by a factor of two. Dispersion impairments after transmission through a 20 km SMF can be observed with a closure of the eye and higher bit-error-rates (BER) as compared to a direct (back-to-back) configuration for both instances. Captured with a similar received optical power (ROP), Figure 4b shows the eye diagrams after propagation through a 20 km SMF and after dispersion compensation using the TE mode of the dispersion compensation device. With 25 Gb/s NRZ data signals, the eye diagram shows a smaller eye opening after transmission through a 20 km SMF due to dispersion impairments as compared to a reopening of the eye after dispersion compensation.

Figure 4c and d shows the bit error rate as a function of the received optical power (ROP) for a direct configuration, after 20 km and after dispersion compensation of a comb line source using the TE and TM mode respectively. It may be observed from Figure 4c that after dispersion compensation is applied to the data signal for transmission of 20 km of SMF, the dispersion penalty is approximately 1 dB as compared to 5.14 dB without dispersion compensation. The dispersion penalty is taken at the BER value of 10^−5^, slightly below commonly used FEC limits [21], [22], [23]. With dispersion compensation in the TE mode, a BER of 3.1 × 10^−11^ could be achieved. It may further be observed from Figure 4c that the BER deviates from the direct configuration less significantly than after the 20 km SMF alone.

To compensate for a 20 km SMF, the normal dispersion region is accessed on the blue side of the grating stopband for the TE polarized light, which has a higher magnitude in normal dispersion value compared to the red side of the stopband. As seen from the group delay spectrum of the TE mode in Figure 2c, the steeper section within the stopband corresponds to a larger magnitude of normal dispersion as compared to the red side at the edge of the stopband. However, operating at the blue side of the stopband comes with a slightly higher insertion loss of ∼3 dB.

By tuning the polarization of the generated frequency comb after modulation, the TM response from the dispersion compensation device can be used, allowing the possibility of dispersion compensation of two polarization states. After transmission of the data through SMF, the light is adjusted for TM polarization prior to coupling into the dispersion compensation device. Figure 4d shows 25 Gb/s NRZ data being modulated on the frequency comb line with a dispersion penalty of 5.5 dB after dispersion compensation as compared to 10.2 dB after 20 km. The dispersion penalty after dispersion compensation with the TM mode is slightly higher as compared to the TE mode. The effect of higher amplification can be seen in Figure 4d with the same pump power of 150 mW, the bit error rates are observed to almost reach an inflection point before a further decrease in BER as ROP is increased. After the inflection point at about −5 dBm, there is a drop in BERs after 20 km and after dispersion compensation where the effects from dispersion compensation becomes more prevalent with a decreasing dispersion penalty at these higher ROPs. A deviation of a straight line also indicates nearing a gain saturation from the EDFA [24].

Figure 4e plots the bit error rates as a function of data rates from 24.33024 to 28.05 Gb/s. We note that these data rates are typically used in commercial transceiver products [22], [23]. In the absence of dispersion compensation after propagation through a 20 km SMF, higher data rates have a much higher BER but significant improvement can be observed after dispersion compensation. With similar ROPs as a comparison, BERs increase as the data rate increases. The BERs achieved after dispersion compensation are below the FEC limit of 10^−5^ and it is observed that the fluctuation in BERs is reduced. At 25 Gb/s NRZ data transmission over 20 km SMF, there is a six order of magnitude improvement in BERs after dispersion compensation to 3.7 × 10^−10^ as compared to 1.4 × 10^−4^ in the absence of dispersion compensation.

### 4.2 All-optical dispersion compensation of PAM4 data after multiple fiber lengths

**Figure 5:**
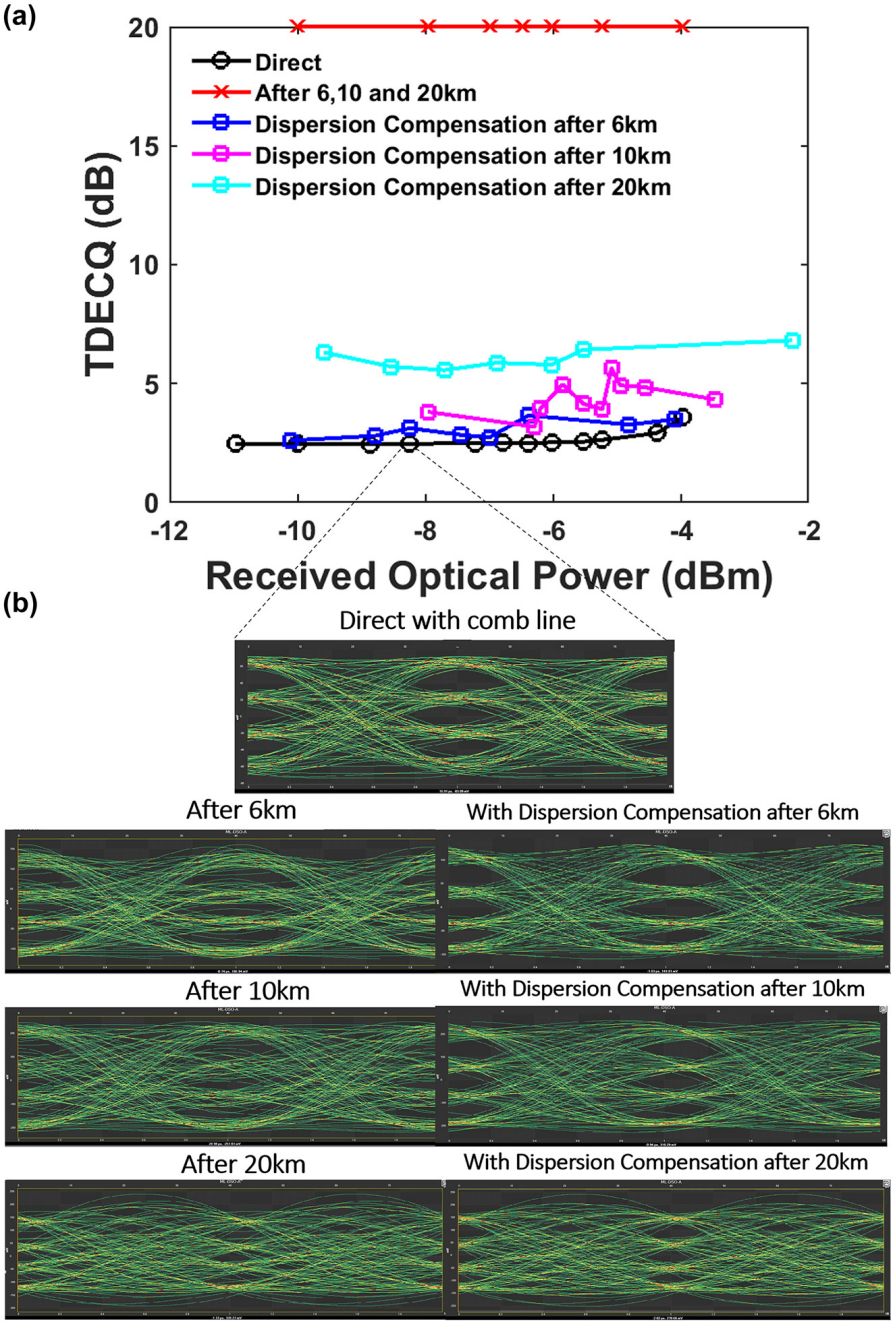
All-optical dispersion compensation at augmented fiber lengths with PAM4 data modulation. (a) TDECQ values as a function of received optical power with and without dispersion compensation after several SMF lengths. (b) Eye diagrams from the transmission of a comb line and the comparisons after transmission through various SMF lengths and after dispersion compensation with 50 Gb/s PAM4 data signals.

We also performed experiments for dispersion compensation of high speed data encoded onto the frequency comb line using PAM4 modulation after propagation through different lengths of SMF. PAM4 modulation has twice the symbol rate as NRZ modulation and thus provides a two-fold increment in the data rate, though at the expense of a −9.54 dB penalty in the signal to noise ratio [25]. Dispersion impairments would therefore be more pronounced when PAM4 modulation is used compared to NRZ modulation. The transmitted data with and without dispersion compensation are shown in Figure 5 for PAM4 data transmitted over a frequency comb line. The improvement with the dispersion compensation device for PAM4 modulation can be quantified by the transmitter and dispersion eye closure quaternary (TDECQ) value [26], [27], [28], shown in Figure 5a, which measures the extent of the optical power penalty where a minimum TDECQ value indicates (=1 dB) an optimal transmitter performance. The TDECQ value increases due to inter-symbol interferences caused by dispersion impairments [29].

Figure 5a shows the TDECQ values for the direct configuration, after various SMF lengths (in red) and after dispersion compensation as a function of ROP. The TDECQ values are averaged over 30 acquisitions for each ROP value. The direct detection configuration indicates the transmission of the frequency comb line with low TDECQ (∼2 dB) as a function of ROP, indicating good SNR which is compliant with the transmitter requirements and near the lower limit for PAM4 data signals as per the multisource agreement 400G-LR4-10. It is observed that the transmission of 50 Gb/s PAM4 data modulated on a primary comb source through 6 km, 10 km and 20 km SMF results in a maximum TDECQ value (20), indicating high signal degradation. A significant improvement in the TDECQ values for all SMF lengths is observed with dispersion compensation. With dispersion compensation, we observe an increase in TDECQ values as the SMF length increases. This shows that an increase in SMF length subsequently introduces noise addition due to the ASE noise from the EDFA to compensate for attenuated power levels. As fiber length increases to 20 km, the required normal dispersion lies within the stopband of the dispersion compensation device, thus also reducing signal transmission and the need for slightly greater amplification at the same received optical power. Figure 5b shows the eye diagrams after propagation of a comb line through various fiber lengths from 6 km, 10 km, and 20 km, with and without dispersion compensation, for the same received optical power. An improvement in the eye opening after dispersion compensation can also be observed in the PAM4 eye diagram for 6 km, 10 km, and 20 km. Therefore, the extent of the dispersion compensation performance relies on a sufficient magnitude of normal dispersion, bandwidth utilization from the dispersion compensation device and the insertion loss where the operating wavelength lies. As a result, our device shows tunable dispersion compensation for SMF lengths varying from 6 to 20 km, for up to 50 Gb/s of data rate.

### 4.3 Dispersion compensation of comb lines at multiple wavelengths

**Figure 6:**
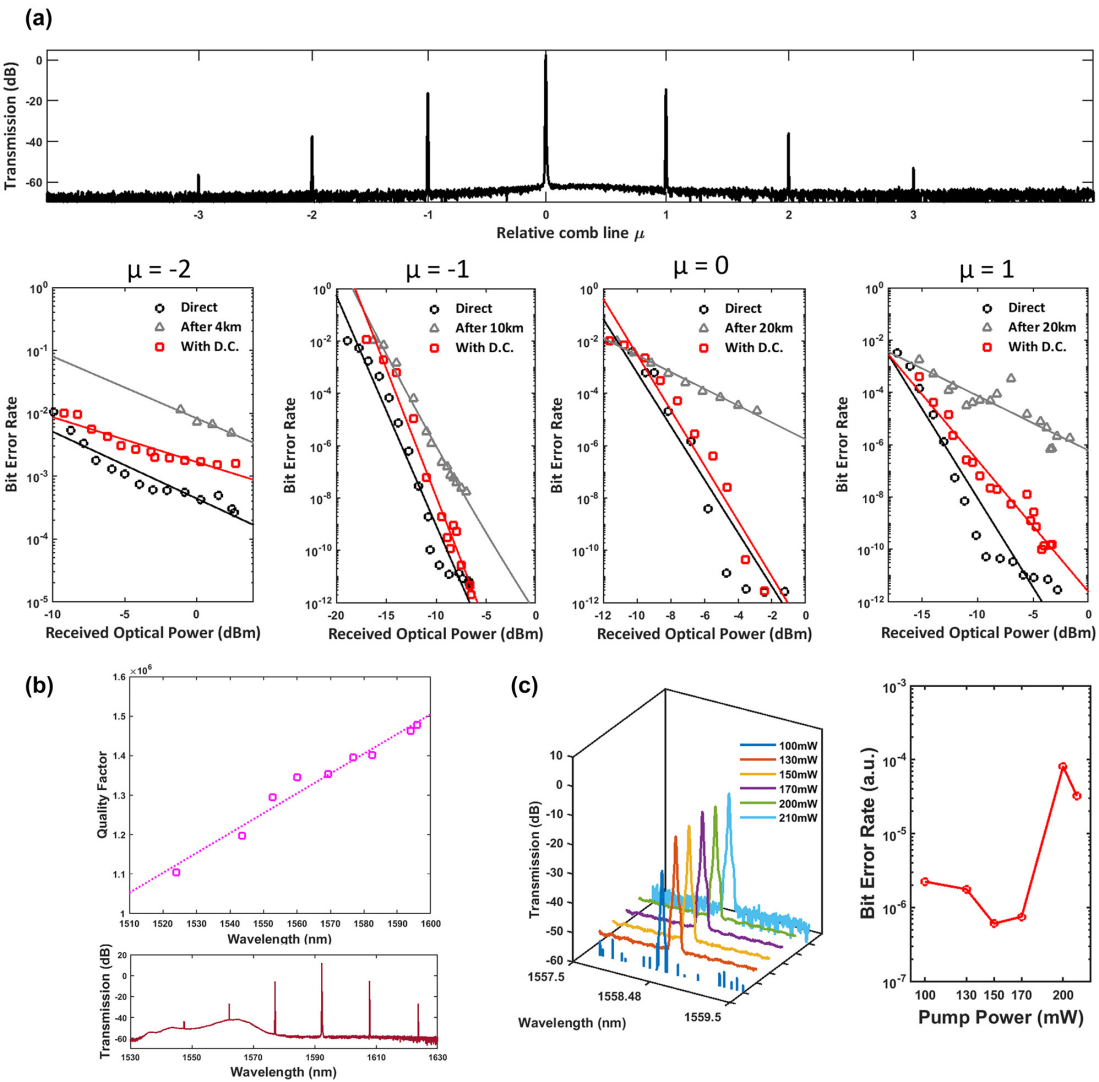
Dispersion compensation across comb lines of a Turing pattern. (a) Dispersion compensation across several comb lines with measured BER as a function of received optical power. (b) Measured loaded quality factor at various resonances from the micro-ring resonator and Turing pattern generated from pump resonance in the L-band. (c) Frequency comb line spectra (left) and high-speed measurement of the bit-error rates (right) as a function of pump power.

We also demonstrate frequency comb generation and dispersion compensation of modulated 25 Gb/s NRZ data signals after 4/10/20 km across the comb lines at different wavelengths from the generated Turing pattern, as shown in Figure 6a. Across the comb lines, it is evident that the dispersion penalty is reduced with the integrated dispersion compensation device as compared to a single-mode fiber alone. For a direct configuration, the BERs for the primary comb lines (relative comb line −1 and +1) is comparatively lower compared to the pump comb line. The generated comb power significantly reduces at relative comb lines of −2 and +2, which requires greater amplification and subsequently leads to higher BERs. At a similar ROP of -8dBm, the BERs for the primary comb lines (μ = −1,+1) are in the region of 10^−11^, 10^−6^ for the pump comb line and 10^−3^ for the outer primary comb lines (μ = −2,+2). The contrast in the BER between the operating wavelength from dispersion compensation of varying fiber lengths of 10 km and 20 km can also be seen with μ = −1, +1 respectively. In all comb lines characterized, a significant improvement in the BER is observed when dispersion compensation with the grating is utilized. Utilizing the red side of the dispersion compensation device in the TE mode, normal dispersion is accessed at the edge of the red side of the bandgap and this accompanies with low insertion loss. However, a larger magnitude of normal dispersion is accessed on the blue side of the TE mode bandgap to compensate for 20 km of SMF, incurring slightly higher insertion loss. For a shorter SMF length of 10 km, the BERs achieved with dispersion compensation follows closely to the direct configuration, indicating the compensation with higher transmission from the dispersion compensation device results in slightly better BERs. The dispersion penalty at a BER of 10^−5^ is 2.4 dB after dispersion compensation and 3.2 dB after transmission through 10 km SMF. Error-free transmission (4.5 × 10^−12^) could be achieved with dispersion compensation when the ROP is −6.5 dBm. After transmission through 20 km SMF, a BER of 10^−11^ can be achieved at approximately −4.5 dBm with dispersion compensation. For the pump comb line (μ = 0), error-free transmission (2.86 × 10^−12^) with dispersion compensation can be achieved at about −2.5 dBm.

The threshold optical power $P_{\text{th}}\propto \left({n_{0}}^{2}/n_{2}\lambda Q^{2}\right)$ , is inversely proportional to the quality factor squared [30], [31]. Figure 6b shows the measured quality factor for various resonances and an increasing quality factor as wavelength increases, indicating a lower threshold optical power to allow the generation of the primary comb state. This grants the use of different pump resonances to generate comb lines extending to the L band for frequency comb generation and data transmission, as shown in the transmission spectrum below in Figure 6b. This extends the flexibility of utilizing more pump resonances to generate more WDM channels.

Lastly, Figure 6c shows the filtered spectra for different pump powers and the BER measured as a function of the pump power with 25 Gb/s NRZ data signals. The BER reaches a minimum with a pump power of 150 mW and sharply increases with a higher pump power from 170 mW to 200 mW. The spectral density increases from 100 mW and remains relatively constant subsequently, but the figure on the right Figure 6c shows that the BER reaches an optimum pump power value. Even with an approximately similar power spectral density from 130 to 210 mW, a higher pump power does not indicate an improvement in the BER as a further increase in pump power could introduce additional noise. As intersymbol interference is inherently linked to dispersion and noise contributions [32], the optimization of the pump power before transmission through a fiber link minimizes intersymbol interference and hence reduces BERs to a certain extent due to the concomitant dispersion and noise contributions.

Furthermore, the optical carrier to noise ratio (power of a comb line to the background noise) is channel dependent with respect to the gain spectrum of the amplifier. From Figure 6a, the location of the frequency comb line at μ = +1 lies near the peak of the EDFA gain spectrum with a lower optical carrier noise ratio, as opposed to at μ = −1 where the comb location is at the tail edge of the EDFA gain spectrum, one would expect a higher SNR with lower ASE noise. Hence, there is a trade-off between the SNR and the power required to optically amplify the signal based on the location of the comb line on the gain spectrum. Requiring more amplification introduces more ASE noise and hence a lower SNR to get an optically high transmission frequency comb line with higher BERs at the receiver end. With an approximate 15 dB reduction in the optical carrier noise ratio, there can be one to two order reduction in BER at lower ROPs. With more amplication spans across in a system, the ASE noise drowns out the noise from the comb line source [33] but as data center communication systems often only have a few spans, the noise contributions from the pump and comb amplification has to also be taken into account. Thus, consideration of the location of the comb line with respect to the gain spectrum is important.

## 5 Discussion and conclusion

In this work, we have successfully demonstrated micro resonator frequency comb based high-speed transmission of IMDD data over long fiber reaches, particularly with the incorporation of integrated dispersion compensation to mitigate dispersion impairments from the optical fiber. The generated primary comb state used in tandem with integrated low loss dispersion compensation allowed us to showcase 25 Gb/s NRZ and 50 Gb/s PAM4 data transmission over up to 20 km of optical fiber. The integrated dispersion compensation at the receiver end of the fiber link shows significant improvement in the eye diagrams with reduced BER up to six orders of magnitude. The device provides low loss, tunable GVD compensation at an estimated latency of around 25 ps. Furthermore, the demonstrated thermo-optic tuning capability of the dispersion compensation device allows compensation of multiple fiber lengths using the same device, enhancing the viability of incorporating on-chip dispersion compensation devices in small form-factor pluggable transceivers. We also demonstrate the flexibility of the dispersive device by utilizing both the TE and TM response for dispersion compensation of high-speed data. This on-chip tunable dispersion feature may potentially be useful for variety of applications which utilize data transmission over fiber, including 5G-enabled applications in Industry 4.0, vehicle-to-everything communication, and internet of things (IoT).

As seen from Table 1, the majority of WDM frequency comb based transmission has been demonstrated with coherent detection formats. It is widely known that the use of DSP has enabled coherent detection and its importance has been shown with long haul transmission communication links. However, this comes with increased complexity and cost as compared to an integrated dispersion compensation solution, which transceivers in data center communication systems are limited by, and why IMDD is still widely deployed. In addition, the latency associated with DSP is typically on the order of a few microseconds [34] compared to ∼25 ps using the dispersion compensation device demonstrated here. We note that the DSP methods with coherent detection in Table 1 are all processed offline. Ref. [4] provided a demonstration whereby IMDD (PAM4) data was modulated on an aluminium–gallium–arsenide-on-insulator microcomb source for PAM4 data transmission over 2 km of fiber. In this work, compensation with DSP methods was performed. While a 2 km fiber reach might not accumulate a significant amount of dispersion impairment, it is useful to note that dispersion compensation might become more important at this reach if the data rate increases, the modulation format becomes more advanced (e.g. PAM8), or the optical carrier noise ratio is lower.

**Table 1:** Comparisons with other WDM microresonator frequency comb-based works.

Microresonator based frequency comb transmission	Format type	Data rate per channel per polarization axis (bit/s)	Maximum fiber length	Dispersion compensation	Achieved BER
[5]	Coherent (16QAM-TDM-PDM-WDM-SDM)	40 Gb/s, 160 Gb/s after OTDM	9.6 km	Offline DSP	2 × 10^−2^ to 10^−4^
[6]	Coherent (64 and 256QAM)	192 Gb/s and 256 Gb/s	7.9 km	Offline DSP with low-density parity-check (LDPC)	<10^−5^
[7]	Coherent (16QAM)	160 Gb/s	75 km	Offline DSP	1.5 × 10^−2^ to 10^−5^
[8]	Coherent (64QAM)	138 Gb/s	76.6 km	Offline DSP	1.67 × 10^−2^ to 3.07 × 10^−2^
[4]	IMDD	64, 80, 100 Gb/s PAM4	2 km	13-tap TDECQ equalizer, 1 dB low-frequency equalization and decision feedback equalization	10^−2^ to 10^−4^
This work	IMDD	25 Gb/s NRZ	20 km	On-chip	10^−12^; TDECQ <6 dB
		50 Gb/s PAM4			

In the work demonstrated here, an on-chip, CMOS-compatible low loss device is used to compensate for dispersion impairments, for data transmission over up to 20 km of SMF. Furthermore, we show the ability to compensate after polarization multiplexing, achieving error-free transmission for 25 Gb/s NRZ data signals and a significant improvement in the TDECQ (>14 dB) for 50 Gb/s PAM4 data signals with the on-chip dispersion compensation device.

Despite the advent of digital signal processing and its capabilities in reducing BERs, the latency introduced by DSP would still warrant mitigation if alternative solutions were available. An on-chip, low-loss dispersion compensation device offers a vastly more compact solution to the fiber link, introducing minimal latency (primarily from the short 4.2 mm device length) as compared to bulkier solutions like dispersion compensating fibers or modules. The possibility of all-optical signal processing whilst achieving low BERs with an on-chip dispersion compensation device is also an attractive substitute with lower complexity and cost. The availability of efficient on-chip devices generating multiple wavelengths of light from a single laser source and the ability to compensate for dispersion through fiber links using integrated photonics facilitates a new paradigm of devices with faster processing speeds and low power consumption.
